# Privacy and Consent in Mobile Health: Solutions for Balancing Benefits and Risks

**DOI:** 10.1016/j.mcpdig.2024.05.005

**Published:** 2024-05-21

**Authors:** Austin T. Gregg, Lisa Soleymani Lehmann

**Affiliations:** a Department of Medicine, Harvard Medical School, Boston, MA; b Department of Health Policy and Management, Harvard T.H. Chan School of Public Health, Boston, MA; c Department of Medicine, Brigham and Women’s Hospital, Boston, MA

Smartphones and associated mobile health (mHealth) applications collect personalized data from millions of users every day. The health information produced creates a mobile medical registry that rivals even the most detailed hospital records. With increasing integration into daily life and the opportunity for machine learning and artificial intelligence (AI) to analyze large volumes of data, mHealth applications have the potential to optimize population health, empower patients, and revolutionize clinical research. However, to fully realize the potential of mHealth, we need to explore the tradeoffs between the benefits and the ethical risks pertaining to privacy and consent. This article aims to provide solutions to strike a balance between the benefits of data sharing at a societal level and individual concerns about data privacy and meaningful informed consent. We propose and discuss differential privacy, dynamic consent, health data commons (HDCs), and research reciprocity for the ethical use of mHealth data.

### Benefits of mHealth

Mobile health promises to make health care more accessible, convenient, insightful, and efficient. Currently, mHealth applications are publicly available within the domains of disease management, medication reminders, fitness, and wellness. For instance, a recent randomized control trial using KENPO-application, a mHealth application that provides specific health guidance for self-monitored weight loss, found a significant reduction (*P*=.03) in body weight in adults with obesity.^1^ Smartphones and their wearable devices are becoming increasingly available as a source of personalized health records. This massive data influx from daily phone usage provides a source of population-based health information that may benefit from machine learning and AI analysis. On a broader scale, usage of mHealth applications is increasing rapidly. The availability of such widespread data has the potential to expedite traditional data collection and expand its reach to populations that have been historically underrepresented in research. Such information can be collected at higher frequencies, noninvasively, and under the user’s daily condition. For example, a recent systematic review found the integration of deep learning, a subset of machine learning, and data from mobile devices facilitated cardiovascular diagnosis, blood glucose prediction, and early cancer detection.^2^ Although still early in its implementation, machine learning and AI may provide insights into a patient health previously believed too difficult to capture accurately.

### Ethical Concerns

There is undoubtedly a need to integrate mHealth data into machine learning algorithms, but doing so may be at odds with ethical concerns about privacy and consent. Users may be apprehensive about the potential for unauthorized access, data breaches, and the misuse of sensitive health information. In the United States, privacy laws, such as the Health Insurance Portability and Accountability Act, protect patient data. Health Insurance Portability and Accountability Act, however, does not extend to mHealth applications. The lack of regulatory standards for security makes it possible for mHealth developers to share a substantial portion of deidentified data, oftentimes without the user’s knowledge. Similarly, the challenges of obtaining meaningful informed consent, although not unique to mHealth, is exacerbated by the ease of clicking yes to a mobile application’s Terms of Use (ToU). By reducing consent to a checkbox operation, it becomes analogous to a transaction rather than an ethically necessary step in patient care.^3^

### What Really Matters to Patients

Despite these ethical concerns, studies have shown that individuals may prioritize the benefits of data sharing over concerns about data privacy and consent. One cross-sectional study of 20,991 mHealth applications found that only 1.3% of users left reviews about privacy concerns.^4^ Although this small percentage may only capture a portion of users with privacy concerns because some may have not felt comfortable leaving negative reviews, this appears to suggest that users may be less worried about the privacy of mHealth applications. Despite the limitations in collecting unbiased reviews, the fact remains that only a small number of users voiced concerns when provided with a platform, albeit imperfect, to do so. In a similar study of the attitudes of 1095 US adults regarding informed consent in medical practices, respondents preferred to be asked for consent but were willing to accept less elaborate approaches if research would otherwise be impractical.^5^ Although consumers may not be amenable to waiving consent altogether, they appear less worried about the rigor with which consent is obtained for data sharing and are agreeable to less stringent forms of consent such as oral consent. Taken together, these findings demonstrate that a low percentage of mHealth consumers report concerns about data privacy and may be willing to trade stringent forms of consent for transparency and accessibility.

### Ethical Solutions to Maximize the Potential Value of mHealth

Solutions are still necessary to further reduce patients’ hesitations and establish a fundamental standard for the ethical use of mHealth as outlined in the Table.^6^, ^7^, ^8^ One solution is to use differential privacy, the use of randomness to safeguard identifiable information, within data sets while allowing useful insights to be extracted. Consider, for example, a mHealth application that wants to estimate the average heart rate of its users over a 12-month period without revealing individual heart rates. To achieve this goal, the application could add carefully calibrated noise to each user’s heart rate before computation. This calculated noise ensures that although the overall trend remains constant, it becomes nearly impossible to pinpoint the exact heart rate of any individual user. Similar methods of differential privacy integration have been used to predict drug sensitivity in cancer treatment, help identify coronary heart disease diagnosis, and analyze functional magnetic resonance imaging.^6^ In fact, investigators found differential privacy to be most commonly used in the fields of genomics, health surveillance with personal devices like mHealth applications, and neuroimaging.^6^ Despite its increasing prevalence in these fields, differentially private processes remains widely underused in mHealth applications.

**Table:** Outline of mHealth Concerns and Proposed Solutions

Problem	Solution	Definition	Example
Privacy of sensitive health data	Differential privacy	Adds calibrated noise to a data set while keeping the overall trend constant	Drug sensitivity prediction models and fMRI imaging analysis^6^
Oversimplified Terms of Use and uncertain meaningful consent	Dynamic consent	Engages users over time to allow for alterations in consent preferences, therefore providing users more granular control	CHRIS, a longitudinal study where users can alter consent preferences and have more granular control of individual data categories^7^
Limited data security	Health data commons	Users can deposit their health information into encrypted research database accessible exclusively by account holder and those given access	MIDATA functions similar to a bank account whereby individuals can securely deposit health information while retaining full control over its utilization^8^
Limited access to users who do not always benefit from data sharing	Data reciprocity	Returns results to users via a symbiotic relationship between researchers and participants	Giving research participants post-trial access to the trial interventions

Abbreviations: CHRIS, cooperative health research in South Tyrol; fMRI, functional magnetic resonance imaging.

Mobile health users are nearly always required to agree to ToU. Typical ToU for commercial applications are a significant departure from the principles of informed consent and an even further departure from the institutional review board clearance of medical research. One solution is capturing dynamic consent. Exemplified by the 10-year longitudinal Cooperative Health Research in South Tyrol study, dynamic consent engages with users over time allowing for alterations in consent preferences. Users can not only choose to opt-in or opt-out, similar to traditional methods of consent, but can also have granular control of individual categories of their data.^7^ This consent can travel with their data so that its scope is never in question. As described by investigators, participants using Cooperative Health Research in South Tyrol dynamic consent found high levels of participation and low change rates from their initial consent preferences but valued the opportunity to change preferences.^7^ Another study using a similar individualized, tiered consent platform in an outpatient clinic found patients preferred granular choices for data sharing. In this setting, most participants consented to share all their data under this platform,^9^ highlighting how higher levels of control may facilitate data sharing.

Next, we recommend mHealth developers use an HDC, a shared platform that consolidates health-related data from various sources to make it accessible for researchers and users. It represents a transformative approach to data management, actualized by companies like MIDATA.^8^ Established by researchers in Switzerland, MIDATA functions more like a secure bank account than a research database, providing individuals the ability to securely deposit their health information while retaining full control over its utilization. Using robust encryption, HDCs can securely store data, accessible exclusively by the account holder. Health data commons empower research participants by collectively crafting a democratic framework for data use. Through this collaborative model, individuals contribute their health data, actively negotiating terms of consent for research. External parties seeking access to these data must submit proposals, subject to scrutiny and approval by the collective, ensuring transparency, privacy, and ethical use.

Finally, reciprocity plays a crucial role in research, fostering a symbiotic relationship between researchers and participants. Although the concept of reciprocity refers to any mutual exchange, research reciprocity emphasizes a return of results to consumers who devote time and effort while accepting risks associated with research. Despite its limited use in medical research, qualitative studies have reported the importance of reciprocity. Specifically, a review analyzing reciprocity obligations in genetic research found that participants felt valued when reciprocity was respected, whereas the absence of reciprocity potentially undermined the mutual partnership and threatened participation in subsequent studies.^10^ Reciprocity in the form of sharing research findings with participants establishes trust, collaboration, and a more inclusive approach to knowledge generation that may ultimately increase users’ willingness to share their health data again.

The massive influx of mHealth data holds incredible promise for making health care more accessible, insightful, and efficient. By implementing solutions that include differential privacy, dynamic consent, an HDC, and research reciprocity, users may be less likely to call privacy into question and more comfortable consenting when they stand to personally gain from sharing their health data. Ultimately, mHealth has the potential to positively impact population health, advance clinical research, and empower patients.

## Potential Competing Interests

The authors report no competing interests.
